# Supplementary material for: A Plant-Derived Antigen–Antibody Complex Induces Anti-Cancer Immune Responses by Forming a Large Quaternary Structure
Source: Int J Mol Sci. 2020 Aug 5;21(16):5603. doi: 10.3390/ijms21165603 (PMC7460599; doi:10.3390/ijms21165603)
Supplement: Supplementary file 1 [file ijms-21-05603-s001.pdf]

International Journal of
Molecular Sciences

A Plant-Derived Antigen–Antibody Complex Induces Anti-Cancer Immune Responses by Forming a Large Quaternary Structure

Deuk-Su Kim, Yang Joo Kang, Kyung Jin Lee, Lu Qiao, Kinarm Ko, Dae Heon Kim, Soon Chul Myeung, Kisung Ko

1. Supplementary Figures

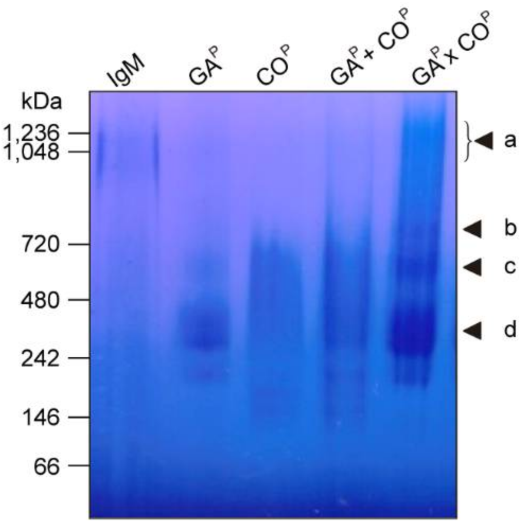

Figure S1. Native-PAGE of IgM, purified sample GA^P, GA^P + CO^P, and GA^P × CO^P. The analysis was performed using native 3–12% gradient polyacrylamide gel stained with Coomassie blue. The a, b, c, and d indicate specific protein bands.

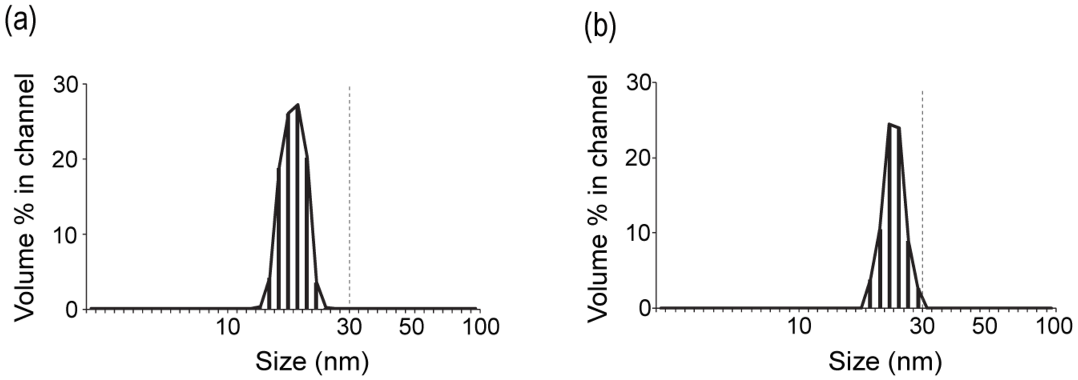

Figure S2. Analysis of size distribution for GA^P and GA^P × CO^P. (a) Size distribution of GA^P were analyzed by dynamic light scattering. (b) Size distribution of GA^P × CO^P was analyzed by dynamic light scattering.

2. Supplementary Material

2.1. Native PAGE analysis

Native-PAGE was performed using a NativePAGE™ Bis-Tris Gel System (InvitrogenTM, Carlsbad, CA) according to the manufacturer's instruction. The purified samples were prepared using a NativePAGE Sample Prep Kit (Life Technologies) and separated on 3–12 % Bis-Tris Gels

(InvitrogenTM, Carlsbad, CA). Native Mark Unstained Protein Standard (LC0725; InvitrogenTM, Carlsbad, CA) was loaded as large molecular protein standards for native-PAGE. The quantitative analysis of purified proteins was determined by a BCA assay (Thermo Fisher ScientificTM, Waltham, MA).

2.2. Dynamic light scattering (DSL)

Dynamic light scattering analyzed the distribution of protein particles by measuring the fluctuating scattered light intensity at a fixed solution. A laser was passed through the sample to measure the particles. The particle diameter was measured using UPA 150 (Microtrac, Montgomeryville, PA). For DLS measurements, 20 μL of GA^P or $GA^P \times CO^P$ protein sample solution was gently mixed with 1,980 μL PBS (1:1,000) to provide a homogeneous solution. About 2 mL of this solution was transferred to a disposable cuvette for particle size measurement. Independent homogeneous solutions were analyzed and three measurements were performed for each reaction.
